# Medicinal Plants Used to Treat Evil Eye Illness in Ethiopia: A Systematic Review

**DOI:** 10.1155/tswj/5498700

**Published:** 2025-05-19

**Authors:** Daniel Tadesse, Getinet Masresha, Moges Lemlemu

**Affiliations:** ^1^Department of Biology, College of Natural and Computational Sciences, University of Gondar, Gondar, Ethiopia; ^2^Department of Plant Sciences, College of Agriculture and Environmental Sciences, University of Gondar, Gondar, Ethiopia

**Keywords:** ethnic background, ethnobotany, evil eye, habit, traditional knowledge

## Abstract

**Background:** Traditional medicinal plants are used worldwide to treat human ailments. One of the ailments used to treat medicinal plants through traditional medical practices is the evil eye. This systematic review aimed to compile and document ethnobotanical studies on the treatment of evil eye illnesses in Ethiopia.

**Methods:** A web-based systematic literature search was performed using electronic databases. All studies with complete ethnobotanical information regarding the plants used to treat the illness were included. A total of 53 articles that met the inclusion criteria were used to compile and document this review. The number of plant species and families, parts used, habits, modes of preparation, and routes of administration was tabulated and summarized using an Excel spreadsheet and descriptive statistics.

**Results and Discussion:** Ethnomedicinal use of 146 plant species belonging to 65 families has been reported and documented for the treatment of evil eye illness in Ethiopia. Fabaceae (22 species) and Asteraceae (13 species) were the most commonly used families in terms of the species count. The dominant habit was shrubs (41%), followed by herbs (31%). Roots (37.13%) and leaves (22.77%) were the first and second most commonly used plant parts, respectively. The most commonly used route of medicinal plant preparation was nasal (35.14%), followed by dermal (22.83%), and oral (18.84%) methods. *Carissa spinarum* L. (Apocynaceae), *Withania somnifera* (L.) Dunal (Solanaceae), *Ruta chalepensis* L. (Rutaceae), *Allium sativum* L. (Amaryllidaceae), and *Capparis tomentosa* Lam. (Capparaceae) are among the most utilized plant species for the treatment of evil eye illnesses.

**Conclusion and Recommendations:** This review shows that many medicinal plants are used to treat evil eye illnesses in traditional medical practices in Ethiopia. Hence, further investigation of commonly utilized plant species for the same purpose is recommended. In addition, the documentation of indigenous herbal knowledge and conservation of commonly utilized traditional medicinal plants should be strengthened.

## 1. Background

In indigenous societies, the diagnosis and interpretation of a health illness mainly relies on supernatural explanations of causative factors, which are strongly attributed to the relationship between the sufferer and his/her surroundings [1, 2]. The evil eye is a supernatural concept.

Dundes [3] defined the evil eye as “a widespread belief in every culture that an individual, male or female, has the power, voluntarily or involuntarily, to cause harm to another individual or his property merely by looking at or praising that person or property.” The common symptoms of evil eye disease include fever, fatigue, lack of appetite, and drowsiness [4]. Harm to the evil eye consists of illness, death, or destruction [3].

Belief in the evil eye dates back to the Stone Age and appears historically and culturally constituted across the globe, including ancient Egyptians, Greeks, and Roman classical writings, as well as in the folklore of Africa, India, China, and other regions [3, 5, 6]. The use of evil eyes is common in many cultures. For example, in most countries, direct, long-held, intense staring by anyone is recognized as an infliction of the evil eye that carries dangerous power [7]. Indigenous healers act as primary healthcare providers because they address specific health concerns with utmost care in many tribal societies [8].

In Ethiopia, those possessing the magical powers of the evil eye are known in the Amharic language as *Buda*. Among the large ethnically and linguistically diverse populations of the Ethiopian highlands and their environs, there are several subtle variations in the central belief in the evil eye, including the Amhara people [9], cattle keepers of the Arusi people [10], Sidamo people [11], Eastern Bench people [12], and the Dorze people [10]. Traditional medicine plays a significant role in the prevention and treatment of evil eyes [13]. Traditionally, medicinal plants (MPs) have been used to treat evil eyes.

A significant amount of research has been conducted in Ethiopia on the ethnobotanical knowledge of MPs used for the treatment of various ailments, including evil eyes. Many of these studies have been compiled and reviewed with a focus on a single ailment. For example, some studies have reported the anti-inflammatory and wound healing activities of MPs [14], potential for cancer treatment [15], treatment of diarrhea [16], erectile dysfunction [17], and liver issues [18].

The lack of previous compilations and reviews of MPs used to treat evil eye illnesses in Ethiopia necessitates the provision of comprehensive ethnobotanical data on these plants. The review questions for MPs used to treat illnesses in Ethiopia were as follows: (1) What types of traditional MPs are commonly used by different ethnic groups in Ethiopia to treat the symptoms associated with the illness? (2) What are the ethnic and regional distributions of the MPs used to treat evil illnesses? (3) Which parts of the MPs are commonly used to treat evil eye illnesses? (4) What is the method of preparing MPs for the treatment of evil eye illnesses? (5) What is the route of administration of MPs used to treat evil eye illness? This systematic review aimed to identify and document traditional MPs used for the treatment of evil eye illness in Ethiopia.

## 2. Methods

The present review of the literature on the major herbal medicines used for the treatment of evil eye illnesses in Ethiopia was conducted using different sources. Relevant information on traditional MPs for the treatment of evil eye illnesses in humans was obtained, and a systematic analysis of indigenous knowledge of different ethnic groups was performed.

### 2.1. Literature Search Strategy

Ethnobotanical studies reporting on MPs used for the treatment of evil eye illness in Ethiopia were identified using international scientific databases, such as PubMed, Science Direct, Web of Science, Scopus, and Google Scholar. Other related websites and PhD dissertation research reports were searched for using Google search engines and university websites.

The key terms used to address the expected objectives were “evil eye illness,” “evil eye,” “medicinal plants,” “traditional medicinal plants,” “indigenous knowledge,” “ethnobotany,” “ethnobotanical study,” and “ethnomedicine.” The search terms were used separately and in combination with Boolean operators such as “OR” or “AND”.

### 2.2. Inclusion Criteria

The inclusion criteria were published and unpublished ethnobotanical surveys reporting MPs related to evil eye illnesses during any period in Ethiopia. The inclusion criteria were restricted to original research articles published in English, which were studied in all parts of Ethiopia. Missing information in some studies (local names, family names, and growth habits of the plants) and misspelled botanical names were retrieved from the Google search engine, botanical databases (Natural Database for Africa Version 2.0, Royal Botanical Garden [Kew], World Flora Online), and Flora of Ethiopia and Eritrea, after which correction/rephrasing was performed.

### 2.3. Exclusion Criteria

Articles lacking basic ethnobotanical information (scientific name, parts used, and route of administration), study areas, or ethnobotanical studies that did not report information on the MPs used to treat the illness were excluded. In addition, studies that included only MPs for livestock usage, non–open access articles, partially accessed (abstract only) articles, review articles, and articles from other countries were excluded.

### 2.4. Study Selection

**Figure 1:**
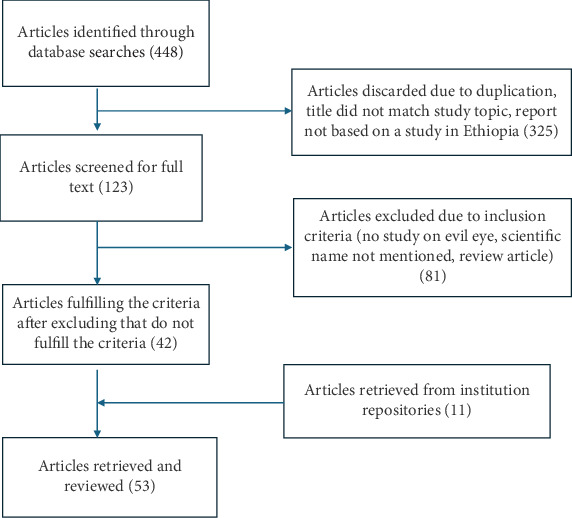
Selection of articles for systematic review.

First, literature screening of the extracted articles involved examining the titles and abstracts of relevant articles for inclusion. Full-text articles were subsequently evaluated using the inclusion and exclusion criteria. The article selection process in this systematic review included 53 studies (Figure 1).

### 2.5. Data Collection

A Microsoft Excel spreadsheet (Microsoft Corporation, United States) was used to collect data on the MP species used to treat acute eye illnesses. The collected data, including botanical name, plant family, local name, part used, growth habits, mode of preparation, and administration, were checked for completeness. A careful review of the articles was performed, and the data were captured using this tool.

### 2.6. Data Analysis

Descriptive statistical methods were used to summarize and analyze the data. The results are expressed as percentages and frequencies and are subsequently presented as tables, bar charts, and pie charts.

## 3. Results and Discussion

Ethnobotanical studies of plants require standard procedures for botanical identification and documentation of indigenous knowledge related to plant distribution, management, and traditional medicinal use in Ethiopia. From an electronic database survey, 53 ethnobotanical studies on MPs used for the treatment of evil eye illnesses in Ethiopia were retrieved.

### 3.1. Ethnic Distribution of MPs

Ethnobotanical studies citing evil eye illnesses included 19 ethnic groups that were distributed throughout the country (Table 1). Considering that the country is home to more than 80 ethnic groups, the illness mentioned by nearly a quarter (23.75%) of the ethnic groups may be related to the low ethnobotanical study coverage. Additionally, respondents' hesitation to mention evil eye illness as one of the ailments of the area treated by MPs may play a part in the lower ethnic representation.

### 3.2. Regional Distribution of MPs

**Figure 2:**
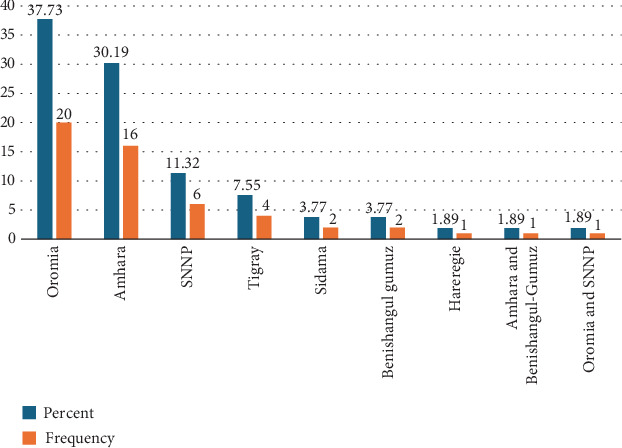
Regional distribution of the studies for treating evil eye illness.

The studies included in this review were conducted in seven regions of Ethiopia, out of the 11 regional states and 2 city administrations (Table 1). The highest proportion of studies was reported in the Oromia region (37.73%), followed by the Amhara (30.19%), and the Southern Nations and Nationalities People (SNNP) region (11.32%) (Figure 2). The regional distribution of the disease showed that this condition was common in several areas and that the locals used traditional healing methods to treat it.

**Figure 3:**
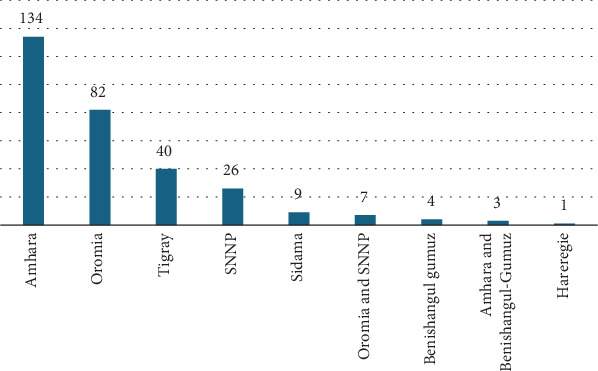
Medicinal plant species mentioned in the regions studied for treating evil eye illness.

Many MP species (134 plant species, 43.79%) have been reported from the Amhara region, followed by the Oromia (82 plant species, 26.79%) and Tigray (40 plant species, 13.07%) regions (Figure 3). The sum of the percentages was greater than 100%, as most MPs used to treat evil eye illnesses are common among regions.

More than 70% of MP species are distributed mainly in the Amhara and Oromia regions, which are among the largest in terms of area coverage and population size. Although diverse ethnic groups and cultural practices are found in the SNNP region, it is the fourth region in terms of distribution of MP species. This may be associated with the fact that only a few studies (six articles) were conducted on this topic compared to the large number of studies conducted in the Oromia and Amhara regions.

### 3.3. Diversity of MPs and Associated Indigenous Knowledge

In the 53 articles reviewed, a total of 146 MP species belonging to 65 botanical families were identified for the treatment of evil eye illnesses (Supporting Information (available here)). This assessment is a good indication of people's deep indigenous knowledge, as it shows how they continue to use indigenous methods to treat evil eye illnesses caused by a wide variety of plant species in Ethiopia. In the supplemental material, each species of MP is described in depth along with its family, local name, habit, plant parts used, method of preparation, and route of administration.

**Figure 4:**
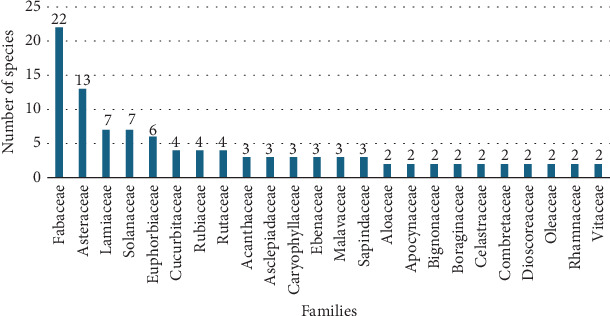
Distribution of species across families.

Fabaceae (22 species) was the most highly represented family, followed by Asteraceae (13 species), Lamiaceae (7 species), Solanaceae (7 species), and Euphorbiaceae (6 species) (Figure 4). These findings are in agreement with those of other reviews [73–75] that showed that Fabaceae is the most represented family. This finding was also consistent with a study conducted in Spain, where Fabaceae and Lamiaceae were the most widely used plant-based rituals for the prevention and treatment of evil eye illnesses [76].

It was found that, in the best-represented family, Fabaceae, which hosts 22 MP species used to treat evil eye illness, biosynthetic pathways produce flavonoids, terpenoids, and alkaloids as secondary metabolites [77–79]. Whether these secondary metabolites play a role in the treatment of this illness requires further investigation.

**Figure 5:**
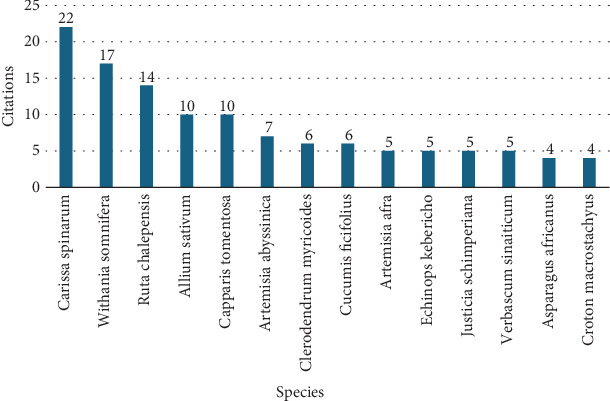
The most cited medicinal plant species used to treat evil eye illness.

The most frequently cited species were *Carissa spinarum* (22), followed by *Withania somnifera* (17), *Ruta chalepensis* (14), *Allium sativum* (10), and *Capparis tomentosa* (10 citations each), as shown in Figure 5. These findings demonstrate that the therapeutic plants indicated above are regarded by the local people as the most effective means of treating evil eye illnesses. On the other hand, the mention of 100 species only once in the reviewed studies showed a wide range of indigenous knowledge exercised to cure ailments (Supporting Information).

These findings agree with those of previous studies in which *Carissa spinarum* [80], *Allium sativum* [76, 81], and *Ruta chalepensis* [76, 82] were used to treat illnesses in various parts of the world. Gonzalez et al. [76] argued that aromatic plants are commonly used to protect individuals from evil eye illnesses. Of the five most frequently cited plant species, *Withania somnifera*, *Ruta chalepensis*, and *Allium sativum* were categorized as aromatic plants, *Carissa spinarum* as an incense plant, and *Capparis tomentosa* as a MP.

### 3.4. Habits of the MPs

**Figure 6:**
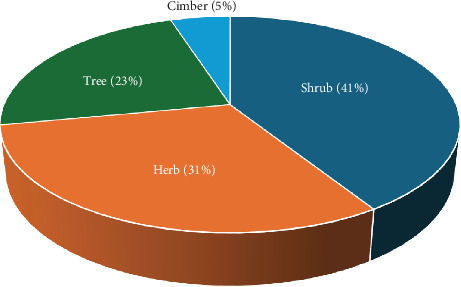
Habit of medicinal plants used to treat evil eye illness.

Assessment of the habits of the MPs used for the treatment of evil eye illness revealed that shrubs constituted the highest fraction, with 60 species (41%), followed by herbs with 45 species (31%), and trees with 34 species (23%) (Figure 6).

Approximately a quarter of the habitat was composed of shrubs and herbs (72%). Therefore, employing more shrubs and herbs could be advantageous because shrubs may be found throughout the year and herbs can be easily cultivated around homesteads. In addition, herbs and shrubs take less time to grow than do trees. However, herbs may be inaccessible during some seasons, particularly if wild sources are used [39, 49]. Several scholars have also reported herbs and shrubs as the most commonly used growth habits for the management of various human and animal ailments in Ethiopia [52, 53, 73, 83].

### 3.5. Utilized Parts of MPs

In this review, several parts of MP species have been reported to be able to treat acute eye illnesses. Roots were the most frequently used (75 citations, 37.13%), followed by leaves (46 citations, 22.77%), and a combination of roots and leaves (15 citations, 7.43%), whereas fruits, seeds, shoots, bulbs, latexes, and flowers were rarely used (Table 2). This review supports the findings of Lulekal et al. [84] and Kefalew et al. [85], who reported that roots are among the most commonly used plant parts in remedy preparations for various disorders in Ethiopia.

One of the advantages of using roots for the treatment of evil eye illness is that fresh roots can be easily harvested throughout the year, as they remain underground even during long dry seasons [49]. However, the extensive use of roots for the treatment of the evil eye may affect the longevity of the plant itself, as has been reported in the literature through the use of roots for the preparation of medications that may endanger MPs [86].

### 3.6. Remedy Preparations

Most of the reviewed articles reported that traditional herbal medicine practitioners usually combine different plant species when preparing herbal medicines for the treatment of eye illnesses. The synergistic interactions that occur among the different phytochemicals in mixed plants may result in increased efficacy. Fumigation is the most common method used for herbal medicine preparation. Others included decoction, infusions, drying, pounding into powder, chewing, and steaming.

### 3.7. Routes of Administration of Herbal Remedies

**Figure 7:**
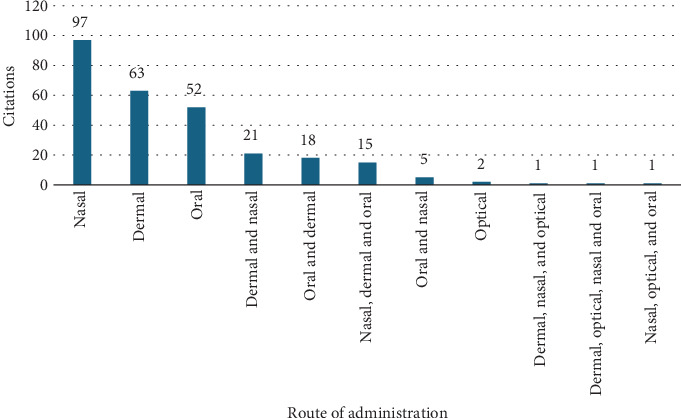
Route of administration of medicinal plants used to treat evil eye illness.

Herbal treatments for evil eye illnesses were delivered nasally (97 citations, 35.14%), dermally (63 citations, 22.83%), or orally (52 citations, 18.84%) (Figure 7). As evil eye is a mental disorder, this review agrees with the MP review on common mental illness in Ethiopia, which states that inhalation is a common route of administration [75].

When using the nasal delivery method, the substance is often smelled rather than directly injected into the nostril. Dermal administration involves tying the neck or arm, smoking the entire body by putting it in fire, brushing the body, or taking a shower while using a decoction. The remedies were also chewed and consumed immediately after collection, or following the first crushing or pounding. The common topical means of administration (nasal and dermal) mentioned in this review may be preferable because they have minimal toxicity and are the simplest and most convenient route of administration.

## 4. Conclusion and Recommendations

In this systematic review, 146 MPs from 65 families were evaluated for use in the treatment of severe eye disease in Ethiopia. These MPs have been used by traditional medical practitioners and local inhabitants in various regions of the country. Fabaceae and Asteraceae are the most widely used families for the treatment of evil eye illnesses. The most frequently cited plant species are *Carissa spinarum*, *Withania somnifera*, *Ruta chalepensis*, *Allium sativum*, and *Capparis tomentosa*. The most common habits were shrubs and herbs. The most utilizable parts of the plants were the roots and leaves.

This analysis showed that Ethiopia has access to numerous traditional MPs from various families that are utilized by many ethnic groups to cure the symptoms of the evil eye. As a result, traditional MPs continue to be important in the community's healthcare system.

This review reveals the widespread occurrence of evil eye disease in Ethiopia, which calls for additional research on commonly reported MPs using modern clinical and scientific methodologies. In addition, it is crucial to prioritize the protection of MPs by safeguarding their natural habitats and encouraging locals to grow them in gardens.

## Figures and Tables

**Table 1:** Characteristics of the studies included in this systematic review.

**No**	**Author and year of publication**	**Study area**	**Ethnic background**	**Number of MPs used to treat evil eye**
1	Misha et al., 2014 [19]	Arsi Negele district, Oromia region	Afan Oromo	1
2	Birhanu and Regassa, 2021 [20]	Dibati district, Benishangul-Gumuz region	Shinasha	1
3	Gebeyehu, 2016 [21]	Mandura district, Benishangul-Gumuz region	Gumuz	3
4	Tebkew et al., 2015 [22]	Chilga district, Amhara region	Amhara	16
5	Hishe, 2019 [23]	Wejig-Mahgo-Waren Massif Forest, Tigray region	Tigre	4
6	Teklehaymanot and Giday, 2007 [24]	Zegie Peninsula, Amhara region	Amhara	11
7	Yineger and Yewhalaw, 2007 [25]	Sekoru district, Oromia region	Afan Oromo	1
8	Hailemariam et al., 2009 [26]	Konta Special district, Southern Nations and Nationalities People region	Konta	1
9	Mesfin et al., 2009 [27]	Wonago district, Southern Nations and Nationalities People region	Gedeoffa	2
10	Teklay et al., 2013 [28]	Kilte Awulaelo district, Tigray region	Tigre	18
11	Megersa et al., 2013 [29]	Wayu Tuka district, Oromia region	Afan Oromo	3
12	Belayneh and Bussa, 2014 [30]	Harla and Dengego valleys, Oromia region	Afan Oromo	2
13	d'Avigdor et al., 2014 [31]	Fiche town, Oromia region	Afan Oromo	2
14	Abera, 2014 [32]	Ghimbi district, Oromia region	Afan Oromo	1
15	Chekole et al., 2015 [33]	Libo Kemkem district, Amhara region	Amhara	22
16	Assefa et al., 2021 [34]	Gura Damole district, Oromia region	Afan Oromo	6
17	Abdela and Sultan, 2018 [35]	Heban Arsi district, Oromia region	Afan Oromo	7
18	Masresha et al., 2015 [36]	Merawi town, Amhara region	Amhara	4
19	Meragiaw et al., 2016 [37]	Delanta district, Amhara region	Amhara	15
20	Alemneh, 2021 [38]	Yilmana Densa and Quarit districts, Amhara region	Amhara	9
21	Tolossa and Megersa, 2018 [39]	Berbere district, Oromia region	Afan Oromo	5
22	Gebeyehu et al., 2014 [40]	Mecha district, Amhara region	Amhara	4
23	Ayele, 2022 [41]	Mojana Wadera district, Amhara region	Amhara	3
24	Regassa, 2013 [42]	Hawassa town, Sidama Region	Sidama	2
25	Teklehaymanot, 2009 [43]	Dek Island, Amhara region	Amhara	3
26	Tolossa et al., 2013 [44]	South Omo Zone, Southern Nations and Nationalities People region	Aari, Maale and Hamer-Bena	3
27	Atnafu et al., 2018 [45]	Girar Jarso and Dagam districts, Oromia region	Afan Oromo	3
28	Yineger et al., 2008 [46]	Bale Mountains National Park, Oromia region	Afan Oromo	6
29	Birhanu and Ayalew, 2018 [47]	Robe town, Oromia region	Afan Oromo	2
30	Enyew et al., 2014 [48]	Fiche district, Oromia region	Afan Oromo	16
31	Getaneh and Girma, 2014 [49]	Debre Libanos district, Oromia region	Afan Oromo	2
32	Giday et al., 2003 [50]	Islands of Lake Ziway, Oromia region	Zay	1
33	Giday et al., 2007 [51]	Dibatie district of Benishangul-Gumuz region and Guangua district of Amhara region	Shinasha, Agew and Amhara	3
34	Giday et al., 2009 [52, 53]	Meinit-Goldya district of Southern Nations and Nationalities People region	Meinit	3
35	Chekole, 2017 [54]	Guba Lafto district, Amhara region	Amhara	7
36	Birhanu, 2013 [55]	Gondar Zuria district, Amhara region	Amhara	1
37	Nigussie and Young-Dong Kim, 2019 [56]	Hawassa Zuria district, Sidama Region	Sidama	7
38	Mengistu et al., 2019 [57]	Haramaya, Babile, and Fedis districts, Hararghe region	Afan Oromo	1
39	Teklehaymanot and Giday, 2010 [58]	Lower Omo River Valley, Southern Nations and Nationalities People region	Kara and Kwego	1
40	Bekele and Reddy, 2015 [59]	Abaya district, Oromia region	Afan Oromo	2
41	Amsalu et al., 2018 [60]	Gozamin district, Amhara region	Amhara	2
42	Wondimu et al., 2007 [61]	Dheeraa town, Oromia region	Afan Oromo	5
43	Masresha et al., 2021 [62]	Metema district, Amhara region	Amhara	7
44	Beyene, 2015 [63]	Erob and Gulomahda districts, Tigray region	Tigre	10
45	Fisaha, 2020 [64]	Menz Gera district, Amhara region	Amhara	16
46	Woldemariam, 2020 [65]	Anlemo, Duna, Gibe, Gombora, and Yem districts, Southern Nations and Nationalities People region	Hadiya, Yem	16
47	Abdurhman, 2020 [66]	Kalu and Bati districts, Amhara region	Amhara	5
48	Alemayehu, 2017 [67]	Gelana district of Oromia region and Amaro district of Southern Nations and Nationalities People region	Afan Oromo, Koore	7
49	Balemie, 2019 [68]	Nole Kaba district, Oromia region	Afan Oromo	4
50	Ashagre, 2017 [69]	Dugda Dawa district, Oromia region	Afan Oromo	7
51	Amsalu, 2020 [70]	Baso Liben and Debre Elias districts of Amhara region	Amhara	9
52	Regassa, 2016 [71]	Jibat, Gedo, and Chilimo forests, Oromia region	Afan Oromo	6
53	Girmay, 2021 [72]	Hirmi woodland vegetation, Tigray region	Tigre	8

**Table 2:** Parts of plants used to treat evil eye illness.

**Parts used**	**Citations/frequency**	**Percentage**	**Parts used**	**Citations/frequency**	**Percentage**
Root	75	37.13	Bulb	2	0.99
Leaf	46	22.77	Root and stem bark	2	0.99
Root and leaf	15	7.43	Stem and leaf	2	0.99
Whole part	13	6.44	Flower	1	0.5
Root bark	11	5.45	Flower and stem	1	0.5
Stem	6	2.97	Latex	1	0.5
Root and stem	5	2.48	Root and latex	1	0.5
Stem bark	5	2.48	Leaf and fruit	1	0.5
Fruit	4	1.98	Root, leaf, and root bark	1	0.5
Seed	4	1.98	Root, leaf, and stem bark	1	0.5
Shoot	4	1.98	Root, leaf, and seed	1	0.5

## Data Availability

All data used in this work are found in the manuscript.

## Supporting Information

Supporting Information Additional supporting information can be found online in the Supporting Information section. The supporting information includes a summary of all the medicinal plants used to treat evil eye ailments in Ethiopia.
