# Supplementary material for: Medicinal Plants Used to Treat Evil Eye Illness in Ethiopia: A Systematic Review
Source: ScientificWorldJournal. 2025 May 19;2025:5498700. doi: 10.1155/tswj/5498700 (PMC12105903; doi:10.1155/tswj/5498700)
Supplement: Supporting Information — Additional supporting information can be found online in the Supporting Information section. The supporting information includes a summary of all the medicinal plants used to treat evil eye ailments in Ethiopia. [file 5498700.f1.docx]

**Supplementary material: Medicinal plants used to treat evil eye ailment**

**Local names:** A: Amharic; Ag: Agew; G: Gumuz; Ge: Gedeoffa; H: Hadya; K: Konta; Ka: Kara; Ko: Koore; Kw: Kwego; M: Meintic; O: Oromiffa; S: Sidama; Sh: Shinashigna; T: Tigray; W: Wolayta; Yem: Yem

**Source:** 1-Abadi Birhanu and Shimels Ayalew (2018); 2-Abebe Ayele (2022); 3-Abesh Birhanu and Tena Regassa (2021); 4-Abiyu Enyew *et al*. (2014); 5-Abraha Teklay *et al*. (2013); 6-Anteneh Belayneh and Negussie Bussa (2014); 7-Balcha Abera (2014); 8-Banchiamlak Nigussie and Young-Dong Kim, Y. (2019); 9-Behailu Assefa *et al*. (2021); 10-d’Avigdor, *et al*. (2014); 11-Derebe Alemneh (2021); 12-Fisseha Mesfin *et al*. (2009); 13-*Gebremicael Fisaha (2020); 14-*Gemedi Abdela and Mustefa Sultan (2018); 15-Gemedo Misha, *et al*. (2014); 16-Genene Bekele, and Reddy, P. (2015); 17-Getinet Masresha *et al*. (20).

| **No** | **Family** | **Scientific name** | **Local name of the plant** | **Habit** | **Plant parts used** | **Method of preparation** | **Route of administration** | **Reference** |
| --- | --- | --- | --- | --- | --- | --- | --- | --- |
| 1 | Fabaceae | *Acacia abyssinica* Hochst.ex Benth. | Girar (A), Memona (T), Hondoddee (O) | Tree | Root | Crush and put on fire for fumigation | Nasal | 10 |
|  |  |  |  |  |  | Crush with garlic, add water | Nasal | 22 |
|  |  |  |  |  |  | Fumigate nasally and body parts | Nasal and dermal | 50 |
| 2 | Fabaceae | *Acacia brevispica* | Kentefa (A) | Shrub | Root | Crush and put on fire for fumigation | Nasal | 45 |
| 3 | Fabaceae | *Acacia etbaica* Schweinf. | Derie (A) | Tree | Root | Put the powder on fire for fumigation | Nasal | 30 |
| 4 | Fabaceae | *Acacia melanoxylon* R.Br. | Omedella (A) | Tree | Root Bark | Grind with *agave sisal* and chew | Oral | 24 |
| 5 | Fabaceae | *Acacia polyacantha* Hochst. ex A. Rich |  | Tree | Root | Crushed and wrap it by a piece of cloth and tie on the neck | Dermal | 43 |
|  |  |  |  |  | Leaf and root | Crush with the leaves of *Withania somnifera*, and then inhaled or burn by fire and fumigated the smoke | Nasal |  |
| 6 | Fabaceae | *Acacia seyal* Del. |  | Tree | Root | Squeezed after crushing with *Allium sativum,* and then put through left nose*.* | Nasal | 43 |
| 7 | Acanthaceae | *Acanthus sennii*  Chiov. | Key kusheshilie (A), kosoru (O) | Shrub | Root | Sniff, Fumigate with fire, Drink | Nasal , dermal and oral | 15 |
|  |  |  |  |  |  | Sniff | Nasal | 52 |
| 8 | Lamiaceae | *Achyrospermum africanum* Hook.f.  ex. Baker | Kebit buda (A) | Shrub | Leaf and root | Chop and soak with water | Oral | 26 |
| 9 | Apocynaceae | *Acokanthera schimperi*  (A.DC.) Schweinf. | Mirez (A), Qaraaruu (O) | Tree | Shoot | Crush and powder | Nasal, optical, and oral | 17 |
|  |  |  |  |  | Leaf and root | crushed with water and squeeze drops | Nasal | 30 |
| 10 | Agavaceae | *Agave sisalana* Perr. | Qacha xale (W) | Shrub | Root | Crush, powder, and drink | Oral | 24 |
| 11 | Lamiaceae | *Ajuga integrifolia* Buch.-Ham. ex D.Don | Zibute-kurijun/qilqilia/qilqilich (M) | Herb | Leaf | Oral | Oral | 34 |
| 12 | Fabaceae | Albizia gummifera (J.F.Gmel.)  C.A.Sm. | Maticho (S), Sensel (A) | Tree | Whole part | Grind, boiling and eat | Oral | 37 |
| 13 | Fabaceae | Albizia  schimperiana Oliv. | Imalaa (O) | Tree | Root | Its root and the root of *Pterolobium stellatum* are dried and powdered | Nasal | 14 |
| 14 | Alliaceae | *Allium sativum* L*.* | Nech shinkurt (A), Shunkurtii  Adii (O) | Herb | Bulb | Grind its bulb with the roots of *Carisa spinarum*, *Phytolacca dodecandra L’ Herit* , *Capparis tomentosa*, *Securidaca longepedunculata.*, *Boscia angustifolia*, *Ruta chalepensis L.*, *Sida schimperiana*, and C*roton macrostachyus*, then inhaling; additionally bandage | Nasal and dermal | 4 |
|  |  |  |  |  |  | Crush its bulb with the roots of *Withania somnifera, Lobelia giberroa, Sida schimperiana,*  *Carissa spinarum, Dodonaea angustifolia, Verbena officinalis, Capparis tomentosa,*  *Croton macrostachyus, Verbasicum siniaticum, Jasminum grandiflorum,*  *Ceratostigma abyssinicum, Clerodendrum myricoides, Ferula communis,*  *Cyphostemma adenocaule*, and *Cucumis ficifolius* and whole parts of *Artemisia afra,*  *Ruta chalepensis*, and *Lepidium sativum* and then sniff the smoke on the burning firewood; the powder form is tied on the neck | Nasal and dermal | 19 |
|  |  |  |  |  |  | Crush, mix with the root of *Solanum incanum* and *Withania somnifera* then add water and drink; | Oral | 47 |
|  |  |  |  |  |  | smelling aroma of bulb | Nasal | 6, 35, 51 |
|  |  |  |  |  |  | Sniff, fumigate with concoction and drink | Nasal, dermal oral | 15 |
|  |  |  |  |  |  | Crushing and  eating | Oral | 20 |
|  |  |  |  |  |  | Smash it bulb with rhizome of *Ginger officinale* and then inhale | Nasal | 27 |
|  |  |  |  |  | Leaf | Crush, heat and pound | Dermal, optical, nasal and oral | 17 |
| 15 | Sapindaceae | *Allophylus abyssinicus* | Embs (A) | Tree | Root | Place on fire and smell | Nasal | 45 |
| 16 | Aloaceae | *Aloe megalacantha*  Bark. | Ere (T) | Shrub | Leaf | Place on fire and fumigate | Nasal | 10 |
| 17 | Aloaceae | *Aloe trichosantha* A. Berger | Wonde- Iret (A) | Herb | Leaf | Chop and cover with polythene bag and tied around neck or waist | Dermal | 30 |
| 18 | Asteraceae | *Artemisia abyssinica* Sch.Bip. ex A.Rich. | Chena baria (T), Chikugn (A) | Herb | Whole part | Mix with bulbs of *Allium sativum* and smell | Nasal | 10 |
|  |  |  |  |  |  | combined with  *Ruta chalepensis, Allium cepa and the* dried skin of  a hyena and put in a pouch of leather as a charm around the neck | Dermal | 13 |
|  |  |  |  |  |  | Put in a pocket as toothbrush; tie its powder with *A. sativum* | Oral and dermal | 19 |
|  |  |  |  |  | Leaf | Crush and sniff | Nasal | 51 |
|  |  |  |  |  |  | Crush and smell as well as tie around the neck | Nasal and dermal | 29, 35 |
|  |  |  |  |  | Root | Crush, put on fire and smoke | Nasal | 45 |
| 19 | Asteraceae | *Artemisia afra*  Jack. ex Willd. | Chikugn (A), Chuqune (O) | Herb | Whole part | Sniff the powder, fumigate and  drink concoction | Nasal, dermal, and oral | 15 |
|  |  |  |  |  | Leaf | Burnt for smoke bath | Dermal | 28 |
|  |  |  |  |  | Leaf | Squeeze with the bulb of *Allium sativum* | Oral and nasal | 30, 41 |
|  |  |  |  |  | Whole part | sniff | Nasal | 35 |
| 20 | Asparagaceae | *Asparagus africanus* Lam. | Kastanito (T), Yesiet kest (A), Hundufana(H), Keshe lzona(Y), Seriti(O) | Shrub | Root | Sniff the powder, fumigate and  drink concoction | Nasal, dermal, and oral | 10, 15 |
|  |  |  |  |  |  | Sprinkle the powder on burning charcoal and smoke | Nasal | 25 |
|  |  |  |  |  |  | Crush, mix in water & wash the body | Dermal | 46 |
| 21 | Melianthaceae | *Bersama abyssinica* Fresen. | Boa(Y), Lolchisa/Tibiro (O), Koreqa(H) | Tree | Shoot | Put nearby | Dermal | 46 |
|  |  |  |  |  | Leaf | Crush and put on the nasal | Nasal | 48 |
| 22 | Fabaceae | *Biancaea decapetala*  (Roth) O. Deg. | Kentefa (A) | Shrub | Leaf and root | Mix with *Ruta*  *chalepensis* and pound it | Oral | 18 |
| 23 | Capparaceae | *Boscia angustifolia* A. Rich | Kermed (A, T) | Tree | Root | Grind the roots of *Carisa spinarum*, *Phytolacca dodecandra*, *Capparis tomentosa*, *Securidaca longepedunculata.*, *Boscia angustifolia*, *Ruta chalepensis*, *Sida schimperiana*, and C*roton macrostachyus*, then inhale and bandage | Nasal and dermal | 4 |
| 24 | Asteraceae | *Bothriocline schimperi*  Oliv.& Hiern ex Benth. | Ulee hare (O) | Shrub | leaf | Sniff | Nasal | 52 |
| 25 | Simaroubaceae | *Brucea antidysenterica*  J.F. Mill. | Haxaawii (O), Abalo (A), Tolo(Y), Chironta(H) | Tree | Leaf | pound and powder | Nasal, dermal, and oral | 17 |
|  |  |  |  |  | Root | Mix with the roots of *Capparis tomentosa* and Carissa *spinarum* and then tie around neck | Dermal | 33 |
|  |  |  |  |  | Root bark | Crush and decoction | Oral | 46 |
| 26 | Loganiaceae | *Buddleja polystachya*  Fresen. | Anfara (O) | Tree | Root bark | Fumigate | Nasal | 52 |
| 27 | Fabaceae | *Caesalpinia decapetala*  (Roth) Alston | Harangama (O) | Tree | Root | Crush | Optical | 17 |
| 28 | Fabaceae | *Calpurnia aurea* (Ait.) Benth. | Chrenchah (T) | Shrub | Leaf | Crush, mix with water and wash the whole body | Dermal | 5 |
| 29 | Capparidaceae | *Capparis tomentosa* Lam. | Gumero (A),  Andel/Harengama (T), Harangama/Gooraa/harangama gurraacha (O) | Shrub | Root | Crash with *Sativum alium, Achyranthes aspera, Temenahe, Ziziphus abyssinica, aliguangua, Ruta chalepensis, Carisa edulis, Clematis simensis, Withtania somnifera, Cucumis ficifolius and Capparis tomentosa* then bandage it | Dermal | 4 |
|  |  |  |  |  |  | Grind with the roots of *Croton macrostachyus*, *Vernonia adoensis*, *Pterolobium stellatum*, and C*arisa spinarum*, then drink the decoction | Oral |  |
|  |  |  |  |  |  | Grind with the roots of *Carisa spinarum*, *Phytolacca dodecandra*, *Securidaca longepedunculata.*, *Boscia angustifolia*, *Ruta chalepensis*, *Sida schimperiana*, and C*roton macrostachyus*, then inhaling; additionally bandage | Nasal and dermal |  |
|  |  |  |  |  | Leaf | Place it on fire for fumigation | Nasal | 10 |
|  |  |  |  |  | Flower | Boil and drink the decoction | Oral | 48 |
|  |  |  |  |  | Root | Drop the infusion into nostrils | Nasal | 49 |
|  |  |  |  |  | Root | Fire fumigates | Nasal  Nasal, dermal, and oral | 30, 15, 35 |
|  |  |  |  |  |  | Sniff the powder, fumigate, and drink concoction |  |  |
|  |  |  |  |  |  | Mix powder paste with water | Oral | 25 |
|  |  |  |  |  |  | Crush and smoke | Nasal | 5 |
|  |  |  |  |  |  | Tie on the neck | Dermal | 38 |
| 30 | Apocynaceae | *Carissa spinarum* L | Sikhua (G), Agam (A),  Hagamsa (O), Atsri (Sh), Mukakerech (M), Egam (T) | Shrub | Root | Crush with garlic and squeeze with water | Oral | 3, 17 |
|  |  |  |  |  |  | Grind, fumigate the smoke and rub the body | Dermal and nasal | 16, 17, 19, 31 |
|  |  |  |  |  |  | Inhale the smoke of the pound | Nasal | 21, 34, 41 |
|  |  |  |  |  |  | Make a juice by mixing with bulb of *Allium* sativum | Nasal | 22 |
|  |  |  |  |  |  | Mix with the roots of *Capparis tomentosa* and *Brucea antidysenterica* and tie around neck | Dermal | 33 |
|  |  |  |  |  |  | Mix with the root of Aloe sp, stem of Clerodendrum myricoides, Croton macrostachyus and Terminalia brownii then fumigate the smoke | Dermal | 47 |
|  |  |  |  |  | Stem | Smoke | Nasal | 42 |
|  |  |  |  |  |  | Fumigate | Dermal | 53 |
|  |  |  |  |  | Leaf | Crush and sniff | Nasal | 51 |
|  |  |  |  |  | Leaf, root and root bark | Chew and take the juice | Oral | 50 |
|  |  |  |  |  | Root | Mix with the roots of Croton macrostachyus, *Capparis tomentosa*, *Vernonia adoensis*, *Pterolobium stellatum* and C*arisa spinarum* then drinking the decoction | Oral | 4 |
|  |  |  |  |  |  | Grind with the root of *Verbasicum sinaiticum, Sativum alium, Achyranthes aspera, Securidaca longepedunculata, Ziziphus abyssinica, Ruta chalepensis, Clematis simensis, Withtania somnifera, Cucumis ficifolius and Capparis tomentosa* then bandage | Dermal |  |
|  |  |  |  |  |  | Grind with the roots of *Phytolacca dodecandra*, *Capparis tomentosa*, *Securidaca longepedunculata.*, *Boscia angustifolia*, *Ruta chalepensis*, *Sida schimperiana*, and C*roton macrostachyus*, then inhaling; additionally bandage | Nasal and dermal |  |
|  |  |  |  |  |  | *Capparis tomentosa* Lam., *Verbascum sinaiticum* Benth., *Achyranthes aspera* L., *Justicia schimperiana* (Hochst. ex A.  Nees) T. Anders  Sprinkling root powder on  burning charcoal and inhaling  smoke | Nasal | 6 |
|  |  |  |  |  |  | *Capparis tomentosa* Lam., *Asparagus africanus* Lam., *Clausena anisata* (Willd.) Benth, *Draceana steudeneri* Engl., *Justicia schimperiana* (Hochst. ex A.  Nees) T. Anders, *Echinops kebericho* Mesfin, *Ruta chalepensis* L., *Allium sativum* L. Sprinkling root powder on burning charcoal and smoke inhaled | Nasal | 6 |
|  |  |  |  |  |  | *Capparis tomentosa* Lam., *Clausena anisata* (Willd.) Benth, Root paste with water and drink | Oral | 6 |
|  |  |  |  |  |  | Crush, mix with whole part of *Withania somnifera* and sulphur and  put it on fire for fumigation | Nasal | 10 |
|  |  |  |  |  |  | Sniff the powder, fumigate, and drink concoction | Nasal, dermal, and oral | 15 |
|  |  |  |  |  | Root bark | Crush with the stem bark of *Ficus sur,* root bark of *Polygala persicariifolia* and *Allium sativum*, then inhale | Nasal | 43 |
|  |  |  |  |  |  | Add the powder on fire and the smoke is allowed to inhale | Nasal | 30, 52 |
| 31 | Celastraceae | *Catha edulis* (Vahl)  Forssk. ex Endl. | Chat (A), Jimma (O) | Shrub | Leaf | Powder | Dermal, nasal, and optical | 17 |
|  |  |  |  |  |  | Mix with water and spit on the body | Dermal | 37 |
| 32 | Lamiaceae | *Chelonopsis moschata* Miq. | Kebit buda (A) | Herb | Leaf and root | Chop and soak with water | Oral | 26 |
| 33 | Chenopodiaceae | *Chenopodium*  *schraderianum* Schult | qoricha gondaa (O) | Herb | Leaf | Sniff the powder | Nasal | 52 |
| 34 | Plumbaginaceae | *Ceratostigma abyssinicum* Asch. |  | Shrub | Root | Tie the powder in the neck and inhale | Dermal and nasal | 19 |
| 35 | Rutaceae | *Clausena*  *anisata* (Willd.)  Benth. | Limich (A) | Shrub | Root | Sniff the powder, fumigate, and drink the concoction | Nasal, dermal, and oral | 15 |
|  |  |  |  |  |  | Crush and sniff | Nasal | 45 |
| 36 | Ranunculaceae | *Clematis longicauda* Steud. ex A. Rich. | Segu(Y), Fiti(O) | Climber | Leaf | Drink the infusion and wash body with the solution | Oral and dermal | 46 |
| 37 | Lamiaceae | *Clerodendrum*  *myricoides*  (Hochst.) Vatke | Lingirtsi (Ag), Misrich/  Misiroch (A) | Shrub | Root | Tie the powder in the neck and inhale | Dermal and nasal | 19 |
|  |  |  |  |  | Leaf | *Mix with the seed of Lepidium sativum and root of Clerodendrum myricoides and then smell, drink or tie around the neck* | Nasal, oral and dermal | 33 |
|  |  |  |  |  | Leaf, root, seed | Crush, powder then  tie on the neck and take with tooth | Dermal and oral | 15 |
|  |  |  |  |  | Root | Put the powder on  burning charcoal and inhale the smoke | Nasal | 25 |
|  |  |  |  |  | Leaf | Crush with *Carissa spinarum* leaf and fire fumigate | Nasal | 30 |
|  |  |  |  |  | Stem and root | Mix with stem of *Terminalia brownie* and fumigate its smoke | Dermal | 47 |
| 38 | Euphorbiaceae | *Clutia*  *Abyssinica* Jaub. & Spach | Fyele fej (A) | Shrub | Root | Place on fire and fumigated by the smoke | Dermal | 44 |
|  |  |  |  |  | Leaf | Place on fire and smoke | Nasal | 45, 48 |
| 39 | Euphorbiaceae | *Clutia lanceolata* Forssk. | Fiyelefej (A) | Shrub | Root | Crush the concoction then tie | Dermal | 35 |
| 40 | Cucurbitaceae | *Coccinia abyssinica* (Lam.) Cogn. | Werq Bemieda (A) | Herb | Root | Crush and eat | Oral | 20 |
| 41 | Nyctaginaceae | *Colignonia ovalifolia* Heimerl | Afesha (A) | Tree | Leaf | Squeeze and inhale | Nasal | 26 |
| 42 | Fabaceae | *Colutea abyssinica* Kunth &  Bouché | Taetaeta (T) | Shrub | Root bark | Tie around the neck | Dermal | 10 |
| 43 | Combretaceae | *Combretum molle*  R.Br. ex G.Don | Rukkensaa (O) | Tree | Root and leaf | Fumigate, drink with camel milk and smell | Nasal, dermal and oral | 50 |
| 44 | Burseraceae | *Commiphora myrrha* Engl. | Qumbi (O) | Tree | Stem | Smoke | Dermal | 29 |
| 45 | Boragnaceae | *Cordia africana* Lam. | Waddissa (Gedeoffa)  Waddesaa (O) | Tree | Root bark | Powdered dry root bark is sprinkled on burning charcoal and smoke is inhaled covered by cloth | Dermal | 9, 40 |
| 46 | Fabaceae | *Crotalaria*  *lachnophora* Hochst.  ex A.Rich. | Qorsa Direyaa (O) | Shrub | Root | Crush, boil & wash | Dermal | 50 |
| 47 | Ephorbiaceae | *Croton macrostachyus* Hochst. Ex. Del*.* | Bisana (A) | Tree | Root | crushed and powdered root is tied on the neck | Dermal | 2, 19 |
|  |  |  |  |  |  | Grind with the roots of *Capparis tomentosa*, *Vernonia adoensis*, *Pterolobium stellatum* and C*arisa spinarum* then drink the decoction | Oral | 4 |
|  |  |  |  |  |  | Grind with the roots of *Carisa spinarum*, *Phytolacca dodecandra*, *Capparis tomentosa*, *Securidaca longepedunculata.*, *Boscia angustifolia*, *Ruta chalepensis*, *Sida schimperiana*, and C*roton macrostachyus*, then inhale and bandage | Nasal and dermal |  |
|  |  |  |  |  |  | Crush, powder then tie on the neck and chew with tooth | Dermal and oral | 15 |
| 48 | Curcurbitaceae | *Cucumis ficifolius* A. Rich*.* | Yemidir Embuay  (A), Sikiya(Y), Wagerecho(H) | Herb | Root and leaf | Crushing and  cooking | Oral | 20 |
|  |  |  |  |  | Root | Crash with the roots of *Carisa spinarum, Sativum alium, Achyranthes aspera, Securidaca longepedunculata, Ziziphus abyssinica, aliguangua, Ruta chalepensis, Clematis simensis, Withtania, and Capparis tomentosa* then bandage | Dermal | 4 |
|  |  |  |  |  |  | Sniff, Fumigate and drink concoction | Nasal, dermal and oral | 15 |
|  |  |  |  |  |  | Root powder is mixed with water and given orally | Oral | 30 |
|  |  |  |  |  |  | crush, decoction, and drink and wash body with decoction | Oral and dermal | 46 |
|  |  |  |  |  | Fruit | Crush, burn and steam | Nasal | 45 |
| 49 | Poaceae | *Cymbopogon martini* | Tej sar (A) | Herb | Leaf and root | Crush, burn and smell | Nasal | 45 |
| 50 | Boraginaceae | *Cynoglossum*  *amplifolium* Hochst.ex  A.Rich. | Qorsa Michi (O) | Herb | Root | Concoct, crush,  powder, mix with  water or milk | Oral | 28 |
| 51 | Vitaceae | *Cyphostemmia adenocaule* Desc. ex Willd & Drimond | Yesha/Agutu (Y),Lalo (H) | Climber | Root | Crush, homogenize with water and wash body | Dermal | 46 |
| 52 | Vitaceae | *Cyphostemma*  *junceum* (Webb)  Desc. Ex Wild & R.B. Drumm. | Etse zewye (T) | Herb | Leaf | Place part on fire for fumigation | Nasal | 10 |
| 53 | Dioscoreaceae | *Dioscorea alata* L. | Boka/awuna (G) | Herb | Root | Place part on fire for fumigation | Nasal | 3 |
| 54 | Dioscoreaceae | *Dioscorea bulbifera* L. | Harae (S) | Climber | Root and leaf | Grinding, chewing, spitting, squeezing | Oral | 37 |
| 55 | Sapindaceae | *Dodonaea angustifolia* L.f. | Kitkita (A), Itacha (O) | Shrub | Root | Crushed, powder and tied on the neck and inhaled | Dermal and nasal | 19 |
|  |  |  |  |  | Leaf | Mix with leaf of *Acokanthera schimperi*, powder and fire fumigate | Nasal | 30 |
|  |  |  |  |  |  | Crush, homogenize with waterand drink | Oral | 48 |
| 56 | Sapindaceae | *Dodonaea viscosa* subsp.  angustifolia (L.f.) J.G.West L.f. | Ittancha (S) | Shrub | Leaf | Boiling, grinding, squeezing, and drinking | Oral | 37 |
| 57 | Flacourtiaceae | *Dovyalis abyssinica*  (A.Rich.) Warb. | Koshimo (O) | Shrub | Root | Concoct, pound,  decoct for steam bath | Dermal | 28 |
|  |  |  |  |  | Stem | Place on fire and fumigate | Dermal | 44 |
| 58 | Dracaenaceae | *Draceana steudeneri* Engl. | Etse Patos/Merqo (A) | Shrub | Root | Burn and inhale the smoke | Nasal | 6 |
|  |  |  |  |  |  | Crush and fumigate | Dermal | 51 |
| 59 | Asteraceae | Echinops longisetus A. Rich. | Qabarichoo (O) | shrub | Root | Burn on fire and inhale its smoke | Nasal | 49 |
|  |  |  |  |  | Root and leaf | The infusion dropped into the eye | Optical |  |
| 60 | Asteraceae | *Echinops hispidus* Fresen. | Keberchoo (O) | Herb | Stem bark | Put on fire and inhale the smoke | Nasal | 11 |
|  |  |  |  |  | Root and stem | Crush and place on fire and inhale | Nasal | 19 |
| 61 | Asteraceae | *Echinops kebericho* Mesfin | Kebercho (A) | Herb | Root | Powder is sprinkled on burning charcoal, and smoke is inhaled | Nasal | 6, 20, 42, 51, 52 |
| 62 | Fabaceae | *Erythrina abyssinica* Lam. ex DC. | Zwawue (T) | Tree | Root bark | Sniff in the fire | Nasal | 53 |
| 63 | Fabaceae | *Erythrina brucei* Schweinf. | Forokocho(Y) | Tree | Stem bark | Crush with *Embelia schimperi, and drink the* concoction and wash | Oral and dermal | 46 |
| 64 | Myrtaceae | *Eucalyptus camaldulensis* Dehnh. | Kelamitos (T) | Tree | Leaf | Sniff on fire | Nasal | 53 |
| 65 | Ebenaceae | *Euclea divinorum* Hiern. | Dedoho (A) | Shrub | Whole part | Crush and tie around the neck | Dermal | 18 |
| 66 | Ebenaceae | *Euclea racemosa* L. | Mi’eesaa (O) | Shrub | Root | Drink the Crushed & decocted and inhaling the smoke | Oral and nasal | 21 |
| 67 | Ebenaceae | *Euclea racemosa*  Murr. subsp. schimperi  (A.DC.) F. White | Keleaw (T) | Shrub | Whole part | Crush and tie powder around the neck | Dermal | 10 |
| 68 | Orchidaceae | *Eulophia streptopetala* Lindl. | Shingurtizibie (T) | Herb | Whole part | Chop with the whole part of *Rumex*  *nervosus and water bath* | Dermal | 53 |
| 69 | Euphorbiaceae | *Euphorbia abyssinica* Gmel | Okma(Y), Adami(O) | Tree | Latex | Added into coffee & drunk | Oral | 46 |
| 70 | Moraceae | *Ficus sur* Forssk |  | Tree | Stem bark | Crush by mixing with root and stem bark of *Carissa Spinarum*, root bark of *Polygala persicariifolia* and *Allium sativum,* then inhale | Nasal | 43 |
| 71 | Apiaceae | *Foeniculum vulgare* Miller | Enslal (A) | Herb | Root and leaf | Pound the powder on fire and snif | Nasal | 23 |
| 72 | Rubiaceae | *Galium boreoaethiopicum* Puff | Mendef adgi (T) | Herb | Root | Placing it on fire for fumigation | Nasal | 10 |
| 73 | Rubiaceae | *Gardenia ternifolia* Schumach. & Thonn. | Gambilo (A) | Shrub | Root and stem bark | Mix with *Allium sativum* powder and  water | Oral and nasal | 22 |
| 74 | Asclepiadaceae | *Gomphocarpus integer* (N. E.  Br.) Bullock | Asaalee-daltii (O) | Herb | Root | Crush and swallow | Oral | 42 |
| 75 | Asclepiadaceae | *Gomphocarpus purpurascens* A.  Rich. | Ari-Yuyo (O) | Herb | Leaf | A cup of Infusion taken | Oral | 12 |
|  |  |  |  |  |  | smoke bath | Dermal |  |
| 76 | Tiliaceae | *Grewia ferruginea* Hochst ex A. Rich | Ogomdii (O, Ge) | Shrub | Root bark | Crush and mix with butter | Oral | 40 |
|  |  |  |  |  | Root bark | Pound with butter and take it | Oral | 9 |
| 77 | Asteraceae | Guizotia scabra (Vis.) Chiov. | Tufoo (O) | Herb | Root | Drink the infusion | Oral | 49 |
| 78 | Acanthaceae | *Hypoestes*  *Forskaolii* (Vahl) R.Br. |  | Herb | Leaf | Add the powder on a fire and fumigate the smoke | Dermal | 44 |
|  |  |  |  |  | Root | Add the powder on a fire and fumigate the smoke |  |  |
| 79 | Fabaceae | *Indigofera spicata* Forssk. | Yebab Alenga (A)  Chaki Aka (Ka) Sharka Nigush (Kw), Gimay, shersherit/sherit/shamtit (M) | Herb | Root | Grind with the roots of *Polygala abyssinica, Carisa spinarum*, *Phytolacca dodecandra*, *Capparis tomentosa*, *Securidaca longepedunculata.*, *Boscia angustifolia*, *Ruta chalepensis*, *Sida schimperiana*, and C*roton macrostachyus*, then inhale and bandage | Nasal and dermal | 4 |
|  |  |  |  |  |  | Chew and insert into nostrils | Oral and nasal | 34, 39 |
| 80 | Oleaceae | *Jasminum*  *grandiflorum* L. | Terhareg (A) | Climber | Root | Sniff, drink and fumigate with concoction | Nasal, oral and dermal | 15 |
| 81 | Cupressaceae | *Juniperus*  *procera* |  | Tree | Stem | Put on fire and smoke the compound | Nasal | 44 |
| 82 | Acanthaceae | *Justicia schimperiana* (Hochst.  ex A. Nees) T. Anders | Sensel/Smiza (A), Dhumuga (O) | Shrub | Root | Smell the aroma of root | Nasal | 6 |
|  |  |  |  |  |  | Smell the aroma of root, fumigate and drink concoction | Nasal, dermal, and oral | 15 |
|  |  |  |  |  |  | Mix with *Carissa spinarum and* put on fire, then sniff | Nasal | 23 |
|  |  |  |  |  | Leaf | Powder and mix with water | Oral | 28 |
|  |  |  |  |  |  | Crush, put on fire and smoke | Nasal | 45 |
| 83 | Asclepiadaceae | *Kanahia laniflora* (Forssk.) R. Br. | Tifrena (A) | Shrub | Latex and root | Crush, drop, and paint | Dermal | 20 |
| 84 | Asteraceae | *Kleinia grantii* (Oliv. &Hiern) Hook.f. | Bierir (T) | Herb | Whole part | Sniff on fire | Nasal | 53 |
| 85 | Asphodelaceae | *Kniphofia pumila* (Ait.) Kunth. | Shingurti zibie (T) | Herb | Bulb | Soak it in water with leaves of Rumex nervosus and wash body with it | Dermal | 10 |
| 86 | Brassicaceae | *Lepidium sativum* L. | Feto (A) | Herb | Seed | Pounding and  drinking | Oral | 20 |
|  |  |  |  |  |  | Mix the powder with water and spray | Dermal | 44 |
| 87 | Fabaceae | *Lens culinaris* Medik. | Msr (A), Define Miser (O) | Herb | seed | Crush, put on fire and smell | Nasal | 45 |
|  |  |  |  |  |  | Eat the seed | Oral | 48 |
| 88 | Campanulaceae | *Lobelia rhynchopetalum* Hemsl. |  | Tree | Root and stem | kept in pocket as toothbrush; the powder is tied with *A. sativum* | Oral and dermal | 19 |
| 89 | Myrsinaceae | *Maesa lanceolata* | Kelewa (A) | Shrub | Root | Crush, burn and smell | Nasal | 45 |
| 90 | Malvaceae | *Malva parviflora* Höjer | Nosma(Y) | Shrub | Leaf | Crush, water homogenate extracted & drunk | Oral | 46 |
| 91 | Celastraceae | *Maytenus arbutifolia*  (Hochst. ex A.Rich.) Wilztek | Qartame/Kom  Bolcha (O) | Shrub | Leaf | Pounded | Oral and nasal | 28 |
| 92 | Caryophyllaceae | *Minuartia filifolia* | Chfrg (A) | Herb | Root | Crush, burn and smell | Nasal | 45 |
| 93 | Cucurbitaceae | *Momordica boivinii* Baill. | Kirae (S) | Climber | Leaf and fruit | Grind, boil,  squeeze and eat | Oral | 37 |
| 94 | Cucurbitaceae | *Momordica foetida*  Schumach. | Kura hareg/Yeamora Genfo (A) | Climber | Root | Crush, put on fire and smoke | Nasal | 45, 51 |
| 95 | Cucurbitaceae | *Mukia maderaspatana* (L.) M.J. Roem. | Gim-Areg (A) | Herb | Root and stem | Chop and tie-on neck or waist | Dermal | 30 |
| 96 | Myricaceae | *Myrica salicifolia* A.Rich. | Nibie (T), Shinet (A) | Tree | Root and stem bark | Tie it on the body, crush and add liquid through the nose | Dermal and nasal | 10 |
|  |  |  |  |  | Root Bark | Crush powder then sniff with the nose | Nasal | 35 |
| 97 | Solanaceae | *Nicotiana tabaccum L.* | Tamboo (O),  Tumako(Y), Tembaa/kosho(H) | Herb | Leaf | Dry, grind, make a powder and then put on fire to fumigate | Nasal | 16 |
|  |  |  |  |  |  | Crush with the leaf of *Croton macrostachyus* &*Vernonia myriantha*& homogenize in water & wash the body of the patient with the solution | Dermal | 46 |
| 98 | Oleaceae | *Olea europaea* L. subsp.  cuspidata (Wall. ex G. Don) Cif. | Woira (A), ejersa (O) | Tree | Stem | Beating with  fresh stick | Dermal | 15 |
|  |  |  |  |  | Leaf | Inhale powder fire smoke | Nasal | 30 |
|  |  |  |  |  |  | Crush and mix with water, the infusion is concoctwith honey or butter and drunk | Oral | 49 |
| 99 | Oliniaceae | *Olinia rochetiana* A. Juss | Nole (S) | Tree | Leaf, Root and stem bark | Grinding, Boiling, Rubbing | Dermal | 37 |
| 100 | Lamiaceae | *Otostegia integrifolia* Benth. | Tungut (A) | Shrub | Stem and leaf | Burn and inhale the smoke | Nasal | 31 |
| 101 | Rubiaceae | *Pavetta abyssinica*  Fresen. | Bootha bekkaa (K) | Shrub | Leaf | Crushed, homogenized in water to drink, and soak the residue the whole body | Oral and dermal | 8 |
| 102 | Piperaceae | *Piper nigrum* L. | Kundo berebere | Herb | Seed | Crush, put on fire and smoke | Nasal | 45 |
| 103 | Pittosporaceae | *Pittosporum*  *viridiflorum* Sims | Irbaa Ykn Budichaa (O) | Shrub | Root and stem | Fumigate and chew | Oral and dermal | 50 |
| 104 | Phytolaccaceae | *Phytolacca dodecandra* L. Herit | Endod (A), Reje (O) | Shrub | Root | Grind with the roots of *Polygala abyssinica, Carisa spinarum*, *Phytolacca dodecandra*, *Capparis tomentosa*, *Securidaca longepedunculata*, *Boscia angustifolia*, *Ruta chalepensis*, *Sida schimperiana* and C*roton macrostachyus*, then inhale and bandage | Nasal and dermal | 4 |
|  |  |  |  |  |  | Crush, homogenize with water | Oral | 48 |
| 105 | Lamiaceae | *Plectranthus igniarius*  (Schweinf.) Agnew | Tonton (S) | Herb | Leaf | Grind, rubb, squeeze, and drink | Oral | 37 |
| 106 | Lamiaceae | *Plectranthus ornatus* Codd |  | Herb | Leaf | Add to fire and fumigate | Dermal | 44 |
| 107 | Podocarpaceae | *Podocarpus falcatus* (Thunb.) R. B. ex. Mirb. | Zigba (T) | Tree | Leaf | Crush, mix with water and wash  the body | Dermal | 5 |
|  |  |  |  |  | Stem | Tie on the neck |  |  |
|  |  |  |  |  | Stem Bark | Crush, mix with *Rumex nervosus*  *and* wash |  |  |
| 108 | Caryophyllaceae | *Pollichia*  *Campestris* Ayton |  | Shrub | Leaf and root | Add on fire and fumigate | Dermal | 44 |
| 109 | Polygalaceae | *Polygala abyssinica* Fres. | Etse libona/Etse-Adin(A) | Herb | Root | Grind with the roots of *Carisa spinarum*, *Phytolacca dodecandra*, *Capparis tomentosa*, *Securidaca longepedunculata*, *Boscia angustifolia*, *Ruta chalepensis, Sida schimperiana*, and C*roton macrostachyus*, then inhale and bandage | Nasal and dermal | 4 |
|  |  |  |  |  | Root | Fire fumigation | Nasal | 30 |
|  |  |  |  |  | Root bark | Crushed with root of *Carissa spinarum,* stem bark of *Ficus sur* and *Allium sativum,* then inhaled | Nasal | 43 |
| 110 | Fabaceae | *Pterolobium stellatum* (Forssk.) Brenan | Keltefa (A) | Shrub | Root | Crushing or  pounding | Nasal | 20 |
| 111 | Fabaceae | *Rhynchosia malacotricha*  Harms | Jidda dhiiga adii (O) | Shrub | Root | Crush and bath | Dermal | 21 |
| 112 | Euphorbiaceae | *Ricinus communis* L. | Shea Qobo (Y) | Shrub | Leaf | Crush, water homogenate, drink, put fresh leaf around the patient | Oral and dermal | 46 |
| 113 | Rubiaceae | *Rubia cordifolia L.* | Kokeku/Kerchicha(Y) | Climber | Leaf | Crush, homogenize in water & wash body parts & drunk its extract | Oral and dermal | 46 |
| 114 | Rosaceae | *Rubus apetalus* Poir. |  | Shrub | Root | Crush and powder smelt | Nasal | 19 |
| 115 | Po1ygonaceae | *Rumex nepalensis* Spreng. | Yewusha Milas (A), Dengele (T) | Herb | Root | Crushing | Oral | 20 |
|  |  |  |  |  | Leaf | Crush, mix with water and wash the whole body | Dermal | 5 |
| 116 | Polygonaceae | *Rumex nervosus* Vahl. | Hehot (T) | Shrub | Leaf | Soak it in water and wash the body | Dermal | 10 |
| 117 | Rutaceae | *Ruta chalepensis* L. | Tena adam (A), Chena adam (T), Charota/  Tena adamii (O), Tsetela (Ko) | Herb | Leaf | Mix with butter, boil, and fumigate | Nasal | **1** |
|  |  |  |  |  |  | Inhale the pound | Nasal | 6, 15, 27, 51, 53 |
|  |  |  |  |  |  | Crush with slices of *Allium sativum,* and tieon the neck | Dermal | 44 |
|  |  |  |  |  |  | drink concoction | Oral | 15, 35, 36 |
|  |  |  |  |  |  | Crush and drink it with boiled *Coffee arabica* | Oral | 10 |
|  |  |  |  |  |  | Crash with roots of *Carisa spinarum Sativum alium, Achyranthes aspera, Securidaca longepedunculata, Ziziphus abyssinica, Clematis simensis, Withtania somnifera, Cucumis ficifolius and Capparis tomentosa* then bandage | Dermal | 4 |
|  |  |  |  |  |  | Grind with the leaf of *Ruta chalepensis*, the roots of *Carisa spinarum*, *Phytolacca dodecandra*, *Capparis tomentosa*, *Securidaca longepedunculata*, *Boscia angustifolia*, *Sida schimperiana*, and C*roton macrostachyus*, then inhale and bandage | Nasal and dermal |  |
|  |  |  |  |  | Fruit | Chew with the bulb of *A. sativum* and stem of *A. afra* | Oral | 19 |
|  |  |  |  |  | Whole part | Crush and  sniff the crushed part | Nasal | 48 |
|  |  |  |  |  | Flower and stem | Pound | Nasal | 20 |
| 118 | Polygonaceae | *Securidaca longepedunculata* Fres. | Temenhie/  Etse menahe  (A), Shitara (T), Xamanaayii (O) | Tree | Root | Grind with the roots of *Polygala abyssinica, Carisa spinarum*, *Phytolacca dodecandra*, *Capparis tomentosa*, *Polygala abyssinica*, *Boscia angustifolia*, *Ruta chalepensis, Sida schimperiana*, and C*roton macrostachyus*, inhale and bandage | Nasal and dermal | 4 |
|  |  |  |  |  |  | Crash with the roots of *Carisa spinarum, Sativum alium, Achyranthes aspera, Securidaca longepedunculata, Ziziphus abyssinica, aliguangua, Ruta chalepensis, Clematis simensis, Withtania somnifera, Cucumis ficifolius and Capparis tomentosa* then bandage | Dermal |  |
|  |  |  |  |  | Root | Crush and put on fire then the smoke sniffed | Nasal | 11, 45 |
| 119 | Fabaceae | *Senna septemtrionalis* (Viv.) Irwin & Barneby | Sinameg/Semamoti(O), Chechoo(H) | Shrub | Fruit | Crush, drink the decoction and wet paste spread over face, head, hand & feet | Oral and dermal | 46 |
| 120 | Fabaceae | *Senna singueana* (Del.) Lock | Gufa (A) | Shrub | Leaf | Mix with *Rumex nervosus* flower and fire fumigate | Nasal | 30 |
| 121 | Malavaceae | *Sida shcimperiana* Hochst.ex A. Rich*.* | Chifrig (A) | Herb | Root | Chewing | Oral | 18 |
|  |  |  |  |  |  | Crush, smoke on the burning firewood and sniff; tie the powder on the neck | Oral and dermal | 19 |
|  |  |  |  |  |  | Grind with the roots of *Polygala abyssinica, Carisa spinarum*, *Phytolacca dodecandra*, *Capparis tomentosa*, *Securidaca longepedunculata.*, *Boscia angustifolia*, *Ruta chalepensis, Sida schimperiana*, and C*roton macrostachyus*, then inhale and bandage | Nasal and dermal | 4 |
| 122 | Malvaceae | *Sida tenuicarpa*  Vollesen | Chifrig (A) | Shrub | Root | As toothbrush and tie on the neck | Oral and dermal | 15 |
| 123 | Sapotaceae | *Sideroxylon*  *oxyacanthum* Baill. | Kombolcha (O) | Shrub | Root | Concoct, pound,  Decoct as steam bath | Dermal | 28 |
| 124 | Caryophyllaceae | *Silene macrosolen* Steud. ex A. Rich. | Waggartii (O),  Wegert (A) | Herb | Root | Grind, make powder and fumigate and rub on the body | Nasal and dermal | 16 |
|  |  |  |  |  |  | inhale the fumigate | Oral | 51 |
| 125 | Asteraceae | *Solanecio angulatus* (Vahl) C. Jeffery | Jiniras (A), Raafuuosolee (O) | Herb | Whole part | Grind, make powder and then boil with oil and drink and then rub on the body | Oral and dermal | 16 |
|  |  |  |  |  | Root | Bath with the powder | Dermal | 21 |
| 126 | Asteraceae | *Solanecio gigas*  Vatke | Yashikoko gomen (A) | Shrub | Root | Sniff, drink and fumigate the concoction | Nasal, oral and dermal | 15 |
| 127 | Solanaceae | *Solanum anguivi* Lam. | Gemekewa(Y) | Shrub | shoot | Smoke by friction & inhale | Nasal | 46 |
| 128 | Solanaceae | *Solanum*  *hastifollium* Hochst.  ex Dunal in DC. | Alalmo kalbi (T) | Shrub | Root | Place it on fire for fumigation | Nasal | 10 |
| 129 | Solanaceae | *Solanum incanum* L. | Yabesha Embuay (A) | Shrub | Shoot | Mix with *Capparis tomentosa* and *Acokanthera schimperi*  leaf, fire fumigate the powder | Nasal | 30 |
| 130 | Solanaceae | *Solanum marginatum* L.f |  | Herb | Fruit | Eat the roasted and pierced ripe fruit with straw or hay fodders | Oral | 19 |
| 131 | Solanaceae | *Solanum schimperianum Hochst. ex A.Rich* |  | Shrub | Leaf | Put on fire and fumigate | Dermal | 44 |
| 132 | Bignoniaceae | Stereospermum kunthianum Cham. | Botoroo (O) | Tree | Stem bark | Put on fire and inhale the smoke | Nasal | 11 |
| 133 | Asteraceae | *Tagetes minuta* L. | Etsefarus (T) | Herb | Leaf | Crush and smell | Dermal | 44 |
|  |  |  |  |  | Whole part | Fumigate and smell | Nasal | 53 |
| 134 | Rutaceae | *Teclea borenensis*  M.Gilbert | Hadheessa (O) | Shrub | Root and leaf | Crushing and drink | Oral | 50 |
| 135 | Bignonaceae | *Tecoma stans* (L.) Juss. | Odnjo or Obraya (G) | Shrub | Root | Crush, diluted in water and drink | Oral | 3 |
| 136 | Combretaceae | *Terminalia brownii* Fresen. | Woyiba (A) | Tree | Stem and leaf | Add on fire and fumigate the whole body | Dermal | 47 |
| 137 | Rutaceae | *Toddalia asiatica* (L.) Lam. | Sego (H),  Yedega-gimero (A) | Shrub | Leaf | Crush, homogenize in water & drink the filtrate | Oral | 46 |
|  |  |  |  |  | Root | Crush with root of Echinops kebericho and liver of Hyena, add water and drink | Oral | 47 |
| 138 | Euphorbiaceae | *Tragia cinerea* (Pax) and Radcl.  Smith | Alebilabit(A) | Herb | Root | Fumigate the fire smoke | Nasal | 30 |
| 139 | Fabaceae | *Trigonella foenumgraecum*  L. | Abish (A) | Herb | Leaf | Crush, add on fire and smell | Nasal | 18 |
|  |  |  |  |  | Seed | Crush, add on fire and smell | Nasal | 45 |
| 140 | Scrophularaceae | *Verbascum*  *sinaiticum*  Benth. | Kutitina (A), Tirnake/handega (T) | Herb | Root | Sniff, drink and fumigate with concoction | Nasal, oral and dermal | 15, 19 |
|  |  |  |  |  | Root | Grind with roots of *Carisa spinarum, Sativum alium, Achyranthes aspera, Securidaca longepedunculata, Ziziphus abyssinica, Ruta chalepensis, Clematis simensis, Withtania somnifera, Cucumis ficifolius and Capparis tomentosa* then bandage | Dermal | 4 |
|  |  |  |  |  |  | Place it on fire with Sulphur for fumigation | Nasal | 10 |
|  |  |  |  |  |  | Crush, burn and steam | Dermal | 45 |
| 141 | Verbenaceae | *Verbena officinalis* L. | Atush (T), Atuch (A) | Herb | Whole part | Put on fire with Sulphur for fumigation | Nasal | 10 |
|  |  |  |  |  | Root | Smell the aroma | Nasal | 6, 15 |
|  |  |  |  |  |  | Drink and fumigate with concoction | Oral and dermal | 15 |
| 142 | Asteraceae | *Vernonia adoensis* Sch. Bip. ex Walp. | Etse Mossie/Mererug (A) | Shrub | Root | Decoct with the roots of Croton macrostachyus *Capparis tomentosa*, *Vernonia adoensis*, *Pterolobium stellatum* and C*arisa spinarum* then drink | Nasal | 4 |
| 143 | Asteraceae | *Vernonia amygdalina* Del. | Girawa (A) | Shrub | Root | Sprinkle the powder on burning charcoal | Nasal | 6 |
| 144 | Solanaceae | *Withania somnifera* (L.) Dunal. | Gizewa (A), Agol (T), Hidi Bude/unso/kumo/Daadoo/  Wahaalee/Lallaaaffa (O) | Shrub | Root | Decoct with the root of *Carisa spinarum* is grind with roots of *Verbasicum sinaiticum, Sativum alium, Achyranthes aspera, Securidaca longepedunculata, Ziziphus abyssinica, Ruta chalepensis, Clematis simensis, Cucumis ficifolius and Capparis tomentosa* then bandage | Dermal | 4 |
|  |  |  |  |  |  | Put the powder in to fire | Nasal | 7, 16, 32, 42 |
|  |  |  |  |  |  | Put the powder on fire, smoke and bandage | Dermal and nasal | 19 |
|  |  |  |  |  |  | Chopp and drink with coffee | Oral | 50 |
|  |  |  |  |  | Whole part | Crush by mixing with roots of Carissa spinarum and put it on for fumigation | Nasal | 10, 13 |
|  |  |  |  |  |  | Crush, mix with *Carissa spinarum*, and then inhale | Nasal | 43 |
|  |  |  |  |  | Leaf | Place on fire and fumigate its smoke | Dermal | 12, 44 |
|  |  |  |  |  | Leaf and root | Crush and drink with water and fumigate with the fume | Oral and dermal | 15 |
|  |  |  |  |  |  | Crush and put on fire then taken smoke | Nasal | 23 |
|  |  |  |  |  | Root | Grind, make powder and wash the body with the prepared powder | Dermal | 16, 46 |
|  |  |  |  |  |  | Drink the decocted and bath with its residue | Oral and dermal | 21 |
|  |  |  |  |  |  | Chop with  garlic and tie on the neck | Dermal | 22 |
| 145 | Rhamnaceae | *Ziziphus abyssinica* Hochst. Ex A. Rich. | Abetere (A) | Tree | Root | Grind with the root of *Carisa spinarum, Verbasicum sinaiticum, Sativum alium, Achyranthes aspera, Securidaca longepedunculata, Ziziphus abyssinica, Ruta chalepensis, Clematis simensis, Withtania somnifera, Cucumis ficifolius and Capparis tomentosa* then bandage | Dermal | 4 |
|  |  |  |  |  |  | Crushed with the roots of *Capris tomentosa* and whole part of *Withania somnifera* then wrapped by piece of cloth, finally tied on the neck or arm. | Dermal | 43 |
| 146 | Rhamnaceae | *Ziziphus spina-christi*  (L.) Desf. | Qurqura/Geba (A) | Shrub | Leaf and root | Fire fumigation | Nasal | 30 |
